# Chromosome-scale genome assembly of a natural diploid kiwifruit (*Actinidia chinensis* var. *deliciosa*)

**DOI:** 10.1038/s41597-023-02006-4

**Published:** 2023-02-14

**Authors:** Hui Xia, Honghong Deng, Mingzhang Li, Yue Xie, Lijin Lin, Huifen Zhang, Xian Luo, Xiulan Lv, Jin Wang, Dong Liang

**Affiliations:** 1 Institute of Pomology and Olericulture, College of Horticulture, Sichuan Agricultural University, Chengdu, 611130 China; 2 Key Laboratory of Breeding and Utilization of Kiwifruit in Sichuan Province, Sichuan Provincial Academy of Natural Resource Sciences, Chengdu, China

**Keywords:** Genome duplication, Plant breeding

## Abstract

The most commercialized kiwifruit, *Actinidia chinensis* var. *deliciosa* (*Acd*), is an allohexaploid (2n = 6x = 174), making high-quality assemblage genome challenging. We previously discovered a rare naturally occurring diploid *Acd* plant. Here, chromosome-level *de novo* genome assembly for this diploid *Acd* was reported, reaching approximately 621.98 Mb in length with contig and scaffold N50 values of 10.08 and 21.09 Mb, respectively, 99.66% of the bases anchored to 29 pseudochromosomes, and 38,990 protein-coding genes and 42.29% repetitive elements annotated. The divergence time of *A. chinensis* cv. ‘Red5’ and ‘Hongyang’ (11.1–27.7 mya) was more recent compared with the divergence time of them and *Acd* (19.9–41.2 mya), with the divergence time of *A. eriantha* cv. ‘White’ being the earliest (22.9–45.7 mya) among that of the four *Actinidia* species. The 4DTv distance distribution highlighted three recent whole-genome duplication events in *Acd*. This is the first high-quality diploid *Acd* genome, which lays an important foundation for not only kiwifruit functional genomics studies but also further elucidating genome evolution of allohexaploid *Acd*.

## Background & Summary

Kiwifruit, belonging to the genus *Actinidia* Lindl. and the Actinidiaceae family (Ericales), is an economically and nutritionally important fruit crop worldwide^1^. China is the world’s largest kiwifruit producer, reaching an annual production of approximately 2.23 million tons, representing slightly more than half of the global annual production (4.34 million tons)^2^. As consumers become increasingly health-conscious, kiwifruit has attracted special attention because it is a nutrient-rich fruit and an ample source of vitamin C, dietary fibers, carotenoids, chlorophylls, and phenols^3,4^. Its nutritional qualities have made kiwifruit known as “the king of the fruits” for centuries^5^.

As sequencing technology and associated bioinformatics tools have advanced, kiwifruit genomic studies have followed suit. To date, four kiwifruit genomes have been published^6^. In 2013, the first draft assembly of *A. chinensis* var. *chinensis* cv. ‘Hongyang’, a diploid heterozygous kiwifruit, was assembled using only short Illumina reads^5^. The genome of *A. chinensis* var. *chinensis* cv. ‘Red 5’ was assembled using short Roche 454 reads^7^. The advent of third-generation sequencing (TGS) technology, such as the Pacific Biosciences (PacBio, Menlo Park, CA, USA) long-read single-molecule real-time (SMRT) sequencing technology, has led to the chromosome-scale genome assembly of *A. eriantha* cv. ‘White’^8^ and an improved genome assembly of ‘Hongyang’, as evidenced by the increased contig and scaffold N50 values^9^.

PacBio is a ‘sequencing by synthesis’ platform similar as to Illumina sequencing, whereas the Oxford Nanopore Technologies (ONT) sequencing, (Oxford Nanopore Technologies, Oxford, UK) is another important TGS method that adopts a nanopore-based single-molecule sequencing strategy. TGS enables the sequencing of very long fragments, up to 30 to 50 kb or even longer. This advantage of TGS has made it a very attractive and suitable option for the high-quality assembly of plant genomes, even if the genomes are extremely large and contain many highly repetitive DNA stretches^10^.

In the genus *Actinidia*, the basic chromosome number is 29, and the ploidy rangs from diploid to hexaploid, and may also be octoploid, decaploid, or dodecaploid^11^. Many species within *Actinidia* bear edible fruits, but four species, including *A. chinensis* Planch. var. *chinensis*, *A. chinensis* var. *deliciosa* (A. Chev.) A. Chev., *A. arguta* (Sieb. et Zucc.) Planch. ex Miq., and *A. eriantha* Benth., are currently cultivated extensively and economically important^1^. An allohexaploid (2n = 6x = 174) *A. chinensis* var. *deliciosa* cv. ‘Hayward’ has been the most popular worldwide and dominated the international market for many years^12^. Green-fleshed kiwifruit like ‘Hayward’ is by now the most commercialized. Despite the importance of *A. chinensis* var. *deliciosa* (*Acd*) in the kiwifruit industry, no genomic information is available for this species. Generating a high-quality *de novo Acd* genome has been challenging because of its high ploidy and large proportion of repetitive DNA sequences.

Over the past few years, most genomic studies have used the diploid *A. chinensis* var. chinensis cv. ‘Hongyang’. Most previous studies on *Acd* have used the hexaploid ‘Harward’, but it only represents one pistillate cultivar from one variety (var. *deliciosa*) belong to one species (*A. chinensis*). The ploidy variation and morphological characteristics of 600 wild populations of *A. chinensis* var. *chinensis* and *Acd* were examined by Li *et al*. (2010), and they found that the majority of *Acd* were classified as tetraploid and hexaploid, with two exceptions being pentaploid and one octoploid^13^.

**Fig. 1:**
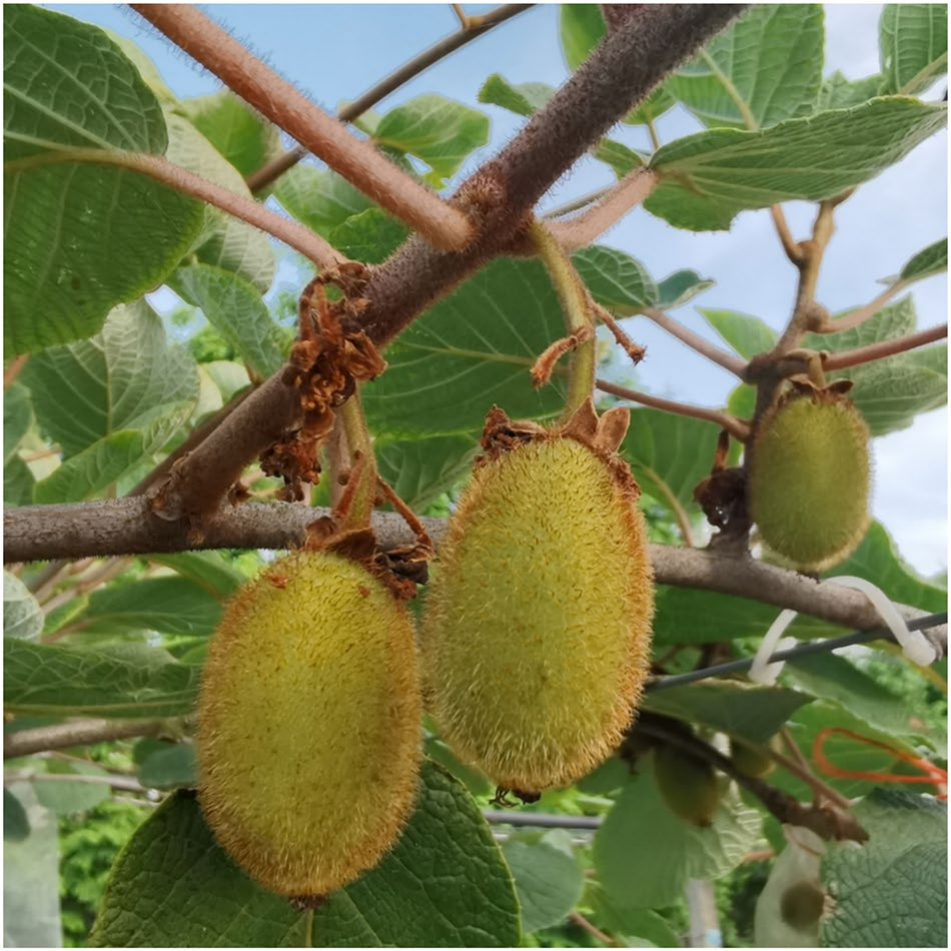
Fruit phenotype of the natural diploid *Actinidia chinensis* var. *deliciosa* resource.

In our kiwifruit breeding program, an unexpected and naturally occurring diploid *Acd* plant was identified and obtained in our previous study (Fig. 1). This is an ideal natural material for unveiling the genomic structure of *Acd*. The benefits of diploid kiwifruit cultivars have also materialized in elucidating the molecular mechanisms underlying multiple development aspects, such as flowering, fruit ripening, and color and flavor development^14–17^. The wild diploid *Acd* plant resource was reported for the first time, which is a rare resource among the natural complex ploidy races in *Acd*. Additionally, wild species may have many important characteristics that can be used to improve the kiwifruit cultivation. Deciphering genomic information is the first step toward maximizing the usefulness of this valuable resource.

**Fig. 2:**
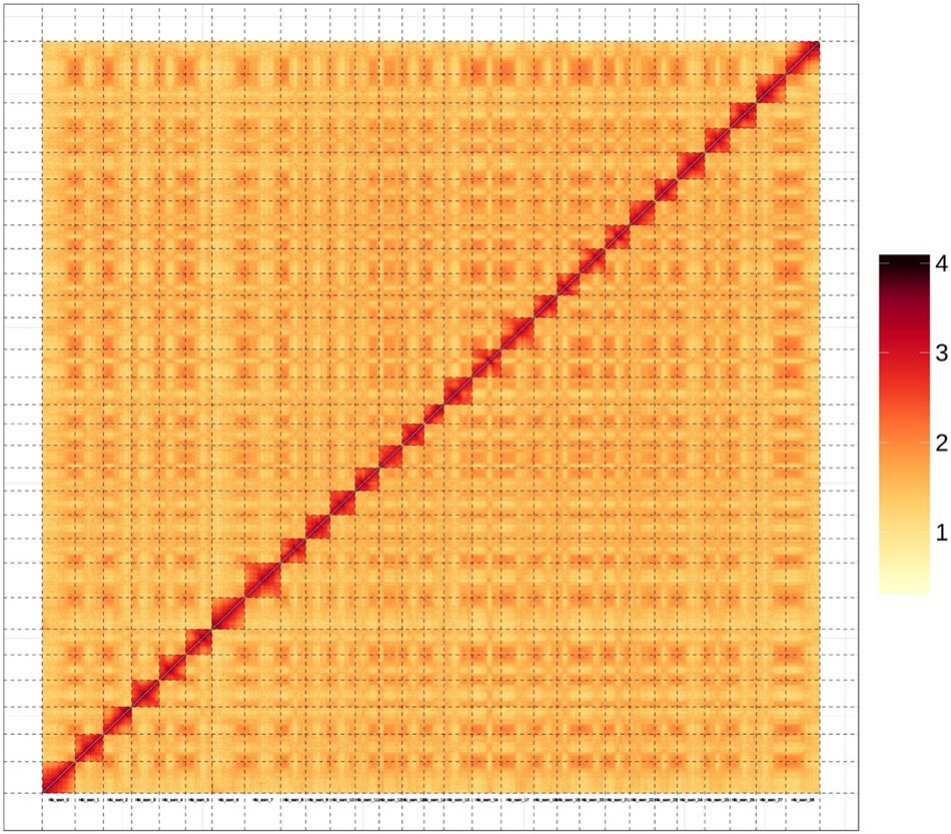
Hi-C interaction heatmap for diploid *Actinidia chinensis* var. *deliciosa* genome. The map shows scaffolded and independently assembled chromosomes at high resolution.

In the present work, a high-quality chromosome-scale genome assembly of the natural diploid *Acd* was constructed by combining ONT sequencing, Illumina sequencing, and chromosome conformation capture (Hi-C) anchoring, which resulted in a genome approximately 621.98 Mb in length with contig and scaffold N50 values of 10.08 and 21.09 Mb, respectively (Table 1). Contigs were scaffolded into 29 superscaffolds, accounting for 99.66% of the total genome size. As shown in the Hi-C heatmap (Fig. 2), the 29 superscaffolds in the *Acd* genome could be distinguished and perfectly represented by 29 chromosomes. The genome assembly presented here is less fragmented and more complete than the four previously published genome assemblies of kiwifruit (Table 1).

**Table 1:** Comparison of the assemblies of *Actinidia chinensis* var. *deliciosa* and the other four published kiwifruit genomes.

Parameter	Actinidia deliciosa	Actiniida eriantha	Actinidia chinensis (v3.0)	Actnidia chinensis (v2.0)	Actnidia chinensis (v1.0)
Total contig length (Mb)	621.98	690.4	653.86	—	604.22
Total contig No.	132	4,076	2,366	—	26,721
Contig N50 (kb)	10,083.34	539.2	1,430	—	58.86
Contig N90 (kb)	3,107.32	50.7	127.9	—	11.6
Longest contig length (Mb)	26.88	3.26	7.8	—	0.42
Total scaffold length (Mb)	621.99	690.6	653.93	548	616.11
Total scaffold No.	41	1,735	1,720	3,887	7,698
Scaffold N50 (kb)	21,085.33	23,583.90	20,000.00	623.8	646.79
Scaffold N90 (kb)	17,895.51	20,112.10	264.7	—	122.7
Longest scaffold length (Mb)	28.56	28.6	27.3	4.43	3.41
Anchored to chromosome (Mb/%)	619.87/99.66	682.4/98.84	640.75/97.98

Comparative genomic analyses were also conducted on the *Acd* genome and other genomes of representative plant species to explore gene and genome evolution. First-ever high-quality chromosome-scale genome for *Acd* was reported in this study. The availability of the genome information provides a critical foundation for not only the phylogeny, genetic diversity, functional genomics, and genomics-assisted breeding studies of kiwifruit but also elucidating genome evolution of allohexaploid *Acd* in future.

## Methods

### Plant materials

Young tender leaf samples were randomly collected from the natural diploid *Acd* plant grown at the experimental base of the Sichuan Provincial Academy of Natural Resource Sciences, Deyang, China (N31°30′, E104°23′). Samples were immediately frozen in liquid nitrogen for Illumina and ONT sequencing. Ploidy was pre-confirmed using flow cytometry. Five types of tissues (leaves, fruit, buds, roots, and branches) were collected and mixed for RNA extraction. A combination of Illumina paired-end (PE), ONT, and Hi-C sequencing was performed by Novogene Co. Ltd. (Beijing, China).

### Illumina sequencing and preliminary genome survey

In order to generate PE genome sequencing libraries with a 350-bp insert size, an Illumina TruSeq® Nano DNA library preparation kit (Illumina, San Diego, CA, USA) was used. The PE libraries were sequenced on an Illumina HiSeq X10 platform using the HiSeq X Ten reagent kit v2.5 (Illumina) for 2 × 150 cycles. Contaminants, low-quality reads, and Illumina adapters were trimmed using “fastp”^18^ prior to downstream genome size estimation. The clean PE reads were subjected to a K-mer counting algorithm using Jellyfish software v.2.2.10^19^. The abundance of 17-K-mer was determined using Kmerfreq v5.0^20^ and provided an estimated genome size of 642.96 Mb with a 0.58% heterozygosity level and a repetitive DNA sequence rate of 49.73%. SOAPdenovo software v.2.0.4^20^ was used for initial assembly. The resutls was then assembled into scaffold using a K-mer parameter of 41, based on the project experience of Novogene, which yielded 2,818 contigs and a scaffold N50 value of 4,791 bp.

RNA extraction and purification were performed using the Qiagen RNeasy Plant Mini Kit (Qiagen, CA, USA), following the manufacturer’s instructions. Equal amounts of RNA extracted from different tissues, including leaves, fruits, buds, roots, and branches, were mixed for RNA-Seq.

### ONT library preparation, sequencing, and assembly

Genomic DNA (10 μg) was mechanically sheared into 10–50 kb fragments using a g-TUBE device (Covaris, Inc., MA, USA). Size selection was performed using the BluePippin size selection system (Sage Science, Inc., MA, USA). DNA end repair was performed using NEBNext End Repari/dA-Tailing Module (New England Biolabs, MA, UK). ONT PromethION sequencing libraries were prepared using the SQK-LSK109 ligation sequencing kit (Oxford Nanopore Technologies) as per the manufacturer’s standard protocol.

After the quality control, NextDenovo software v.2.3.0 (https://github.com/Nextomics/NextDenovo) was applied to correct the error rate of Nanopore long reads using the Illumina short reads. The preliminary assembly was further polished by Illumina PE reads using the NextPolish software v.1.2.4. The redundant contigs led by heterozygosity were identified and removed using Purge Haplotigs. After filtering low-quality reads, 80.32 Gb of Illumina short-read and 79.0 Gb of ONT long-read data were obtained, representing approximately 124.92- and 122.87-fold coverage of the estimated genome (642.96 Mb), respectively.

### Hi-C assisted scaffolding

Hi-C library preparation and sequencing followed the protocol of Belton *et al*. (2012)^21^ with minor modifications. The chromatin was cross-linked with a 4% formaldehyde solution. The cross-linked nuclei were digested using restriction enzymes targeting GATC and GANTC restriction sites. A biotin-labeled nucleotide was incorporated at the digested ends, and the ends were ligated. Ligation products were purified and prepared to an appropriate size (150 bp) for Illumina short-read sequencing. The Hi-C reads were linked into pseudochromosomes using ALLHiC software v.0.9.14. After ALLHiC scaffolding, Juicebox v1.13.01 was used to manually correct large-scale inversions and translocations to obtain the final pseudochromosomes.

### Repetitive element identification

An alignment of homologous sequence and a *de novo* prediction method were combined to identify repetitive elements. The former adopted the RepeatMasker v.4.1.0 and Repbase library (http://www.girinst.org/repbase/) to identify known transposable elements (TEs), which were then aligned with the genome sequences from a TE protein database, RepeatProteinMask (http://www.repeatmasker.org/). The latter applied the LTR_FINDER v.1.0.6 (http://tlife.fudan.edu.cn/ltr_finder/), RepeatScout v.1.0.5 (http://www.repeatmasker.org/), and RepeatModeler v.2.0.1 (http://www.repeatmasker.org/RepeatModeler.html) to construct a *de novo* repeat library, and then adopted the RepeatMasker v.4.1.0 to predict the repetitive element family.

In total, 42.29% of the assembled sequences were identified as repetitive DNA sequences. The most abundant repetitive DNA sequences were retrotransposons (35.85%), of which the long terminal repeat (LTR) retrotransposons accounted for 35.55% with Gypsy (13.99%) and Copia (13.23%) elements being dominant. The DNA transposons represented 0.62% of the genome (Table 2).

**Table 2:** Repetitive elements and their proportions in diploid *Actinidia chinensis* var. *deliciosa* genome.

Repeat family	*De novo* + Repbase		TE proteins		Combined Tes	
	Length (bp)	Proportion in genome (%)	Length (bp)	Proportion in genome (%)	Length (bp)	Proportion in genome (%)
DNA transposons	3,819,086	0.61	126,403	0.02	3,881,658	0.62
LINE	1,865,614	0.3	140,320	0.02	1,915,779	0.31
SINE	10,314	0	0	0	10,314	0
LTR	########	34.87	46,054,847	7.4	########	35.55
Unknown	30,012,173	4.83	0	0	30,012,173	4.83
Total (TRF not included)	########	40.06	46,321,434	7.45	########	40.58

### Protein-coding genes prediction

The protein-coding genes were annotated by incorporating transcriptome data, homology-based searches, and *de novo* predictions, resulting in 38,990 genes. In the homology-based prediction, we used TblastN v.2.2.26 (http://blast.ncbi.nlm.nih.gov/Blast.cgi) and GeneWise software v.2.4.1. Augustus v.3.2.3^22^, Geneid v.1.4^23^, Genescan v.1.0^24^, GlimmerHMM v.3.04^25^, and SNAP v.2013.11.29^26^ were used for *de novo* prediction. *De novo* transcriptome assembly was performed using Trinity v.2.1.1^27^. Hisat v.2.0.4^28^/TopHat v.2.0.11^29^ was used to align RNA-Seq reads to the genome FASTA to identify the exons and splice sites. Stringtie v.1.3.3^30^/Cufflinks v.2.2.1 was used to assemble transcripts based on the alignment results. To generate a consensus gene set, we used EvidenceModeler (EVM) v.1.1.1 and the Program to Assemble Spliced Alignments^31^ to merge genes predicted in the three respective annotation files.

The average transcript length and coding sequence size were 5,457.64 and 1,178.37 bp, respectively, with a mean of 5.28 exons per gene. The average exon and intron lengths were 223 and 1,000 bp, respectively. The number of annotated genes in *Acd* was close to that in the *A. chinensis* v3.0 genome, which has 40,466 genes (Table 3).

**Table 3:** Comparison of the gene structure of the diploid *Actinidia chinensis* var. *deliciosa* and other published kiwifruit genomes.

Species	Number	Average transcript length(bp)	Average CDS length(bp)	Average exons per gene	Average exon length(bp)	Average intron length(bp)
*Actinidia chinensis* var. *deliciosa*	38,990	5,457.64	1,178.37	5.28	223.26	1,000.28
*Actiniida eriantha*	42,988	4,809.50	1,006.73	5.07	198.72	935.22
*Actinidia chinensis (v3.0)*	40,466	4,900.83	1,027.86	5.3	193.91	900.55
*Actnidia chinensis (v2.0)*	33,124	5,140.63	1,278.43	5.47	233.78	864.3

### Gene function annotation

Functional annotation of the predicted genes was performed by aligning them to SwissProt, NCBI non-redundant protein (Nr), Kyoto Encyclopedia of Genes and Genomes (KEGG), InterPro (https://www.ebi.ac.uk/interpro/), Gene Ontology (GO), and protein families (Pfam). A search of publicly available databases (ProDom^32^, PRINTS^33^, Pfam^34^, SMART^35^, PANTHER^36^, and PROSITE^37^) was conducted using InterProscan v.5.27.66 to annotate the motifs and domains. A total of 38,079 genes (97.70% of the predicted protein-coding genes) were annotated using the above multiple databases. Specifically, approximately 78.00%, 96.60%, 75.30%, 94.70%, 59.10%, and 74.10% were annotated in SwissProt, Nr, KEGG, InterPro, GO, and Pfam, respectively.

### Non-coding RNA annotation

Prediction of transfer RNAs (tRNAs) was conducted using tRNAscan-SE v.1.3.1 (http://lowelab.ucsc.edu/tRNAscan-SE/). We used BLAST to predict rRNAs based on the conservation of plant rRNA sequences. Other non-coding RNAs (ncRNAs), including microRNAs (miRNAs) and small nuclear RNAs (snRNAs), were identified using Infernal software v.1.1.2 (http://infernal.janelia.org/) by searching against the Rfam v14.1 database. The predicted non-coding genes included 5,626 ncRNAs, including 967 miRNAs, 705 tRNAs, 1,042 rRNAs, and 2,912 snRNAs in the *Acd* genome.

### Comparative genomics analysis

**Fig. 3:**
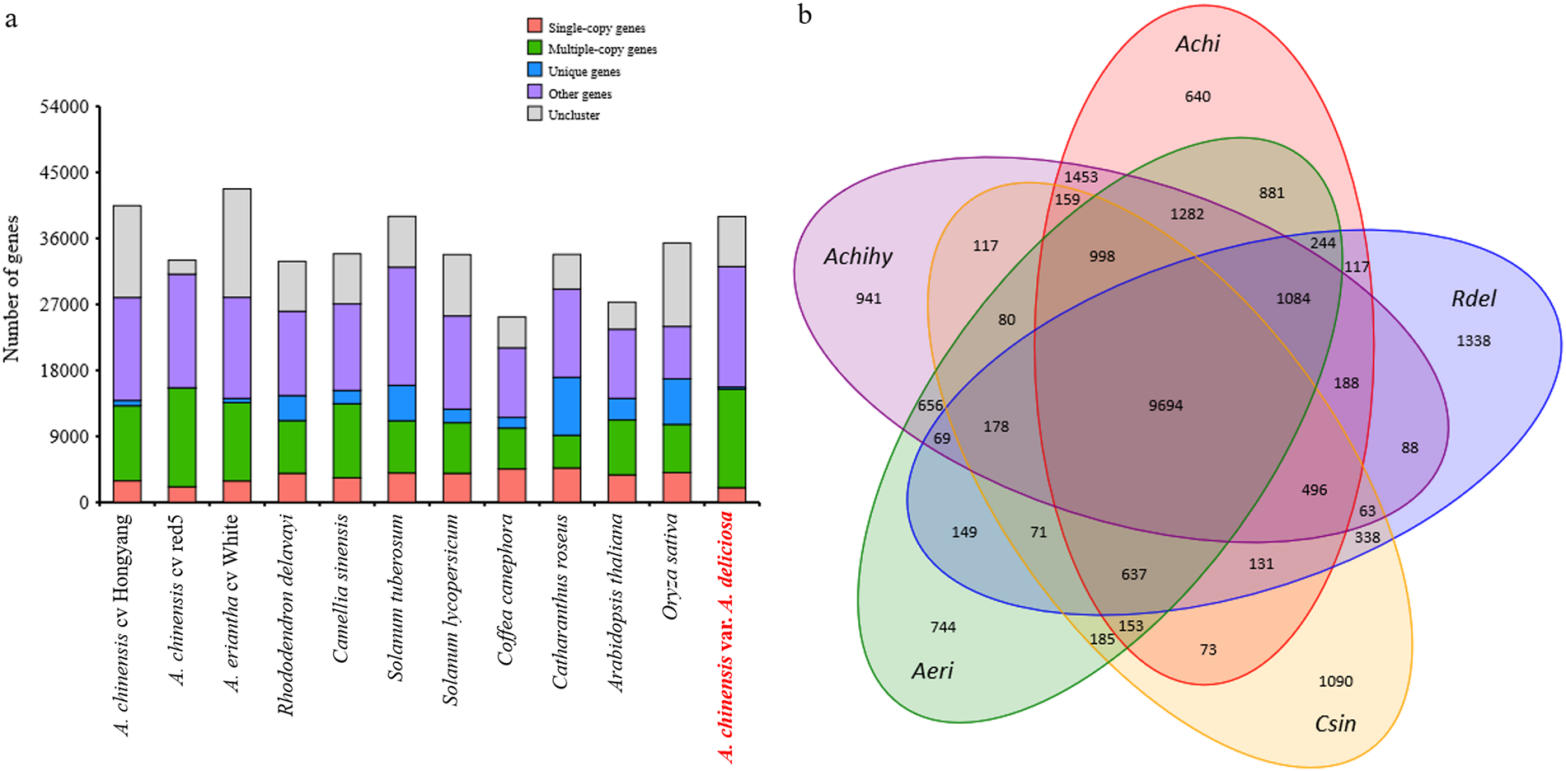
Gene distribution in *Actinidia chinensis* var. *deliciosa* and 11 other representative species (**a**) and the common and unique gene families in *Actinidia chinensis* var. *deliciosa* and four other species (**b**). The single-copy genes and multiple-copy genes are presented in all the 12 plant species. The unique genes are only presented in one of these 12 plant species and the other genes are those presented in two to eleven plant species.

Orthologous relationships between the genes of the currently assembled genome of *Acd* and other 11 representative plant species, including *A. chinensis* cv. ‘Hongyang’, *A. chinensis* cv. ‘Red5’, *A. eriantha* cv. ‘White’, *Rhododendron delavayi*, *Camellia sinensis*, *Solanum tuberosum*, *Solanum lycopersicum*, *Coffea canephora*, *Catharanthus roseus*, *Arabidopsis thaliana*, and *Oryza sativa*, were analyzed through all-against-all protein sequence similarity searches using OrthoMCL software v.2.0.9 (http://orthomcl.org/orthomcl/). For each orthologous cluster, we retained only the longest predicted transcript per locus. Clustering of these 12 plants’ protein-coding sequences yielded a total of 32,884 gene families. There were 6,159 common gene families and 694 common single-copy gene families (Fig. 3a), which represent the evolutionarily conserved and ancestral gene families. In *Acd*, 816 genes assigned to 640 gene families were unique (Fig. 3b).

The alignment was generated using Muscle v3.8.31 (http://www.drive5.com/muscle/) and was further refined using Gblocks 0.91b (http://molevol.cmima.csic.es/castresana/Gblocks.html). Based on protein sequences from single-copy orthologous groups, a maximum likelihood phylogenetic tree was constructed using RAxML v.8.2.12 (http://sco.h-its.org/exelixis/web/software/raxml/index.html). *Acd* was placed as a sister clade to the cultivated *A. chinensis* species (Red 5 and Hongyang), indicating its closer relationship with the *A. chinensis* than *A. eriantha*. Additionally, phylogenetic results showed that *Actinidia* species were most closely related to *R. delavayi* and *C. sinensis*. The divergence time with 95% confidence intervals among these 12 plant species was calculated using MCMCtree. The calibration points for divergence time estimation were taken from the TimeTree website (http://www.timetree.org/). The divergence time was as follows: *R. delavayi* - *A. chinensis*, 52.0–96.0 million years ago (mya); *C. canephora* - *C. roseus*, 61.0–95.0 mya; *S. tuberosum* - *S. lycopersicum*, 5.23–9.40 mya; *S. tuberosum* - *A. thaliana*, 111.0–131.0 mya; and *C. canephora* - *O. sativa*, 115.0–308.0 mya. The divergence time of the *A. chinensis* cv. ‘Red 5’ and *A. chinensis* cv. ‘Hongyang’ (11.1–27.7 mya) was more recent compared with the divergence time of *A. chinensis* cv. ‘Red 5’ and *A. chinensis* cv. ‘Hongyang’ and *Acd* (19.9–41.2 mya), with the divergence time of *A. eriantha* cv. ‘White’ being the earliest 22.9–45.7 mya among that of the four *Actinidia* species.

**Fig. 4:**
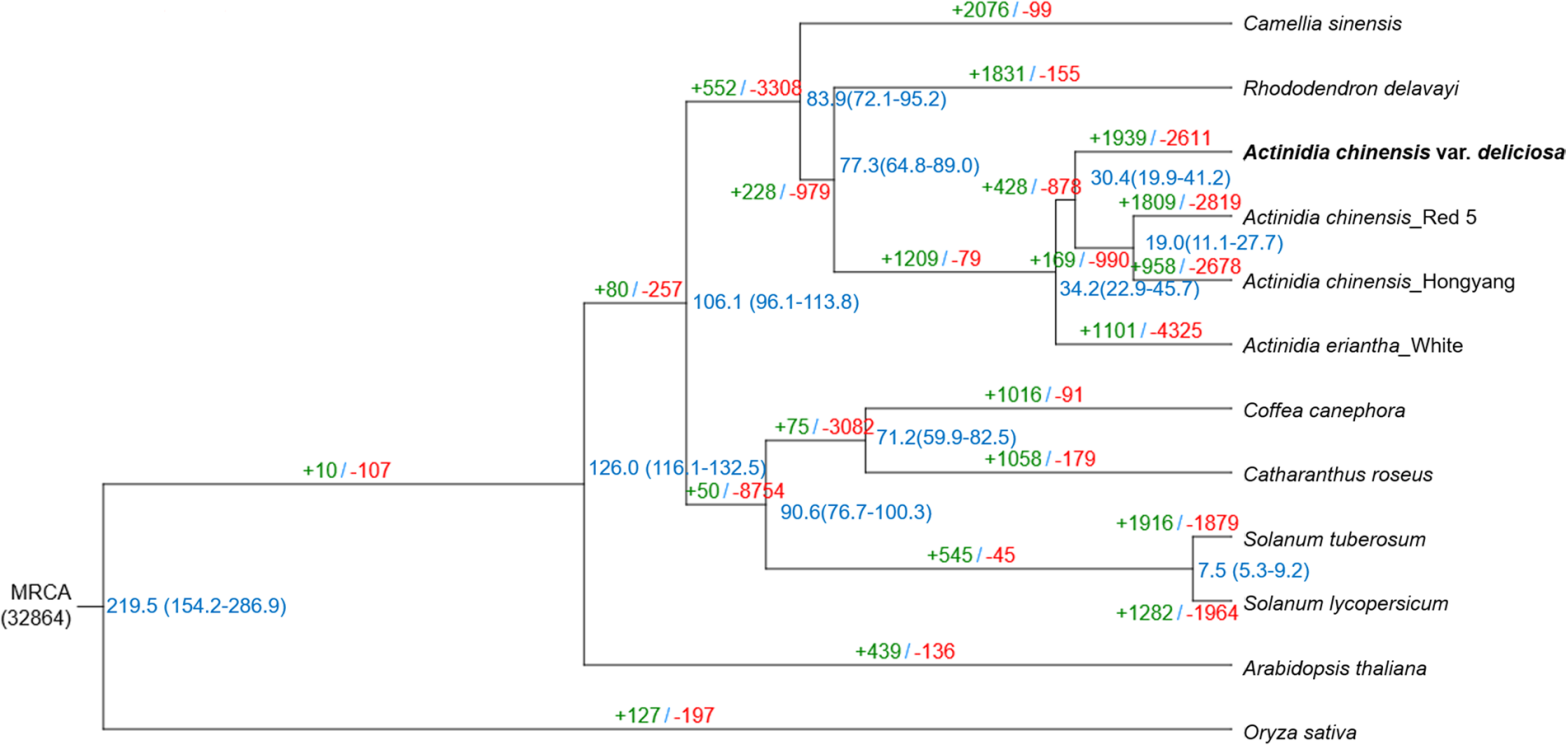
Phylogenetic tree of the 12 species, and gene family expansion and contraction. Inferred divergence times are denoted at each node in blue. Gene family expansion and contraction are indicated in green and red, respectively.

The maximum likelihood tree was used as a starting point for estimating species divergence time using the MCMCtree program (http://abacus.gene.ucl.ac.uk/software/paml.html), which was incorporated into the Phylogenetic Analysis using the Maximum Likelihood (PAML) software v4.9e. The expansion and contraction of the gene families were determined using Computation Analysis of gene Family Evolution (CAFE) v3.1 (http://sourceforge.net/projects/cafehahnlab/). In total, 1,939 and 2,611 gene families expanded and contracted in the *Acd* genome, respectively (Fig. 4).

### Synteny and whole-genome duplication analysis

**Fig. 5:**
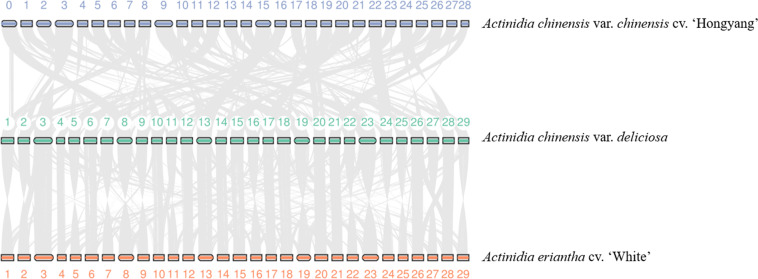
Genome synteny among *Actinidia chinensis* var. *deliciosa*, *A. chinensis* cv. ‘Hongyang’ and *A. eriantha* cv. ‘White’.

The homologous proteins between *Acd* and *A. chinensis* cv. ‘Hongyang’, *A. chinensis* cv. ‘Red5’, and *A. eriantha* cv. ‘White’ were identified using BLASTP. MCScanX^38^ was used to assess the synteny between genomes of *Acd* and *A. chinensis* cv. ‘Hongyang’, *A. chinensis* cv. ‘Red5’, and *A. eriantha* cv. ‘White’, with at least five syntenic genes and no more than 15 gapped genes. Genome synteny analysis revealed a higher degree of synteny between the genomes of *Acd*, *A. chinensis* cv. ‘Hongyang’, and *A. eriantha* cv. ‘White’ (Fig. 5).

**Fig. 6:**
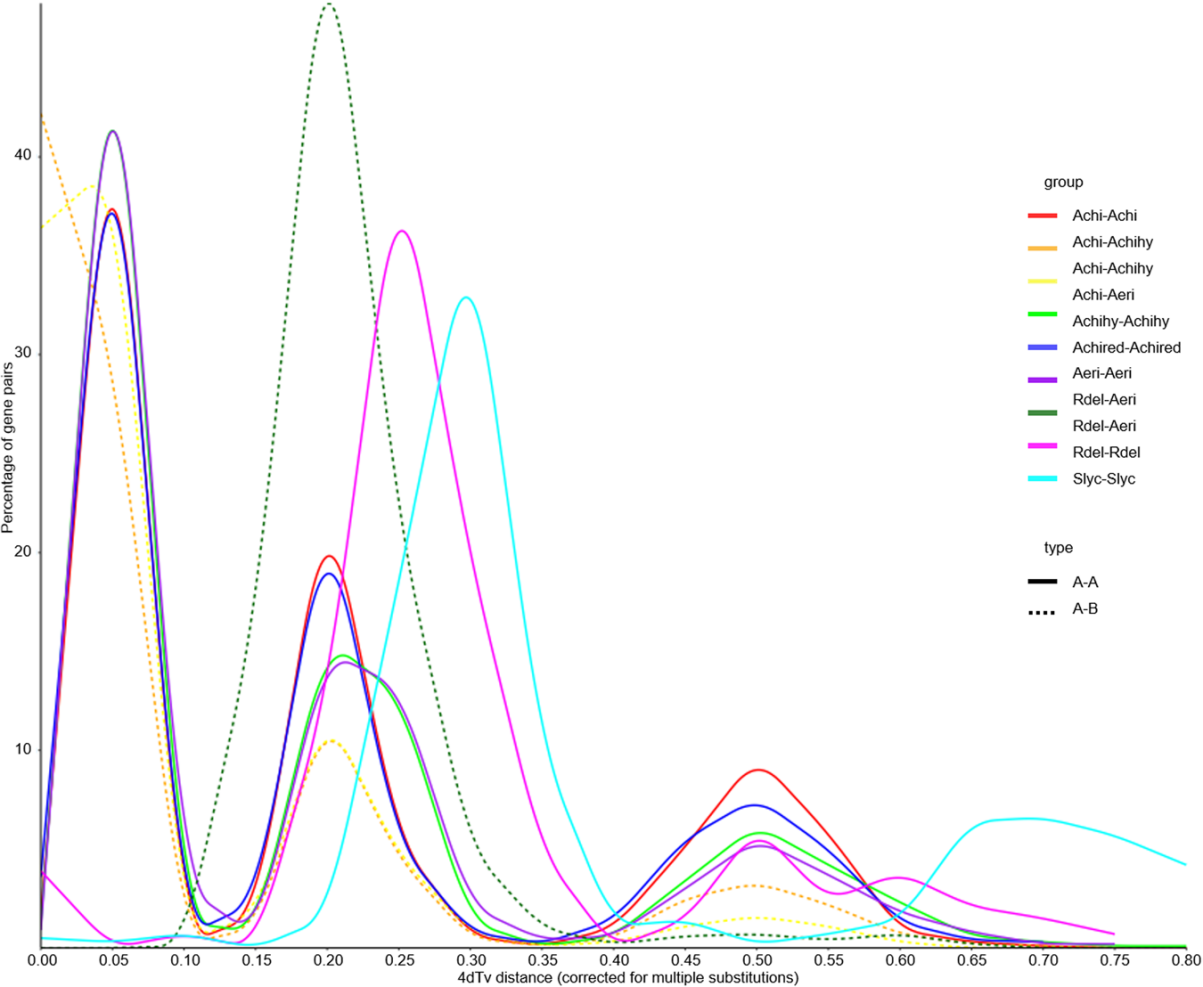
Distribution of the 4DTv rates among the paralogs of the studied species. The dashed line shows the species divergence event, and the solid line shows the duplication event.

As a neutral genetic distance, four-fold degenerated sites (4DTv) has been widely used to analyze evolutionary divergence and estimate the relative timing of putative whole-genome duplication (WGD) events. The 4DTv value peaked at 0.05, 0.2, and 0.5, highlighting three recent WGD events in *Acd*. The speciation event that gave rise to the *Actinidia* species, represented by the pairwise 4DTv distribution of *A. eriantha* cv. ‘White’ against *R. delavayi*, occurred almost simultaneously with the duplication event found in *Actinidia* species (Fig. 6).

## Data Records

The genome sequencing data have been deposited in the NCBI database under the project ID PRJNA850524^39^ and SRX17463538^40^. The genome estimation, assemble statistics based on 41-K-mer, assemble statistics based on Illumina and Nanapore sequencing data, BUSCO assessment, GEGMA assessment, Illumina reads coverage, single nucleotide polymorphisms, repetitive elements, gene function annotations, ncRNA prediction, gene families, gene expansion, and gene contraction have been deposited at the Figshare database with 10.6084/m9.figshare.20279286.v2^41^.

## Technical Validation

The completeness of the final assembled genome was assessed using Benchmarking Universal Single-Copy Orthology (BUSCO) software v4.0.5 by searching plant-specific databases that contain a total of 1,440 orthologous single-copy genes. The results revealed the retrieval of 96.1% of the complete single-copy genes, of which 27.8% were duplicated. Only 0.7% of BUSCO genes were fragmented, and 3.2% were missing from the genome. The BUSCO results indicated high genome assembly completeness.

Core Eukaryotic Genes Mapping Approach (CEGMA, v2.0) employs 248 most highly conserved core eukaryotic genes (CEGs) to assess the extent of comprehensive gene coverage. The genome assembly showed a high completeness level, with 95.16% (236) of CEGs completely and partially covered, including 81.05% (201) of CEGs considered complete.

The filtered short Illumina reads were aligned back to the genome assembly using Burrows–Wheeler Aligner (BWA) software v.0.7.12-r1039 (http://bio-bwa.sourceforge.net/). Approximately 99.25% of the short reads mapped to the genome, and 98.72% were marked as properly paired. The ratios of heterozygous and homozygous single nucleotide polymorphisms (SNPs) were 0.422941% and 0.000588%, respectively, indicating that the assembly had high single-base-level accuracy.

## Data Availability

NextDenovo software v.2.3.0: read_cutoff = 1k, seed_cutoff = 26,457, sort_options = -m 20 g -t 10 -k 40, minimap2_options_raw = -x ava-ont -t 10, correction_options = -p 10, minimap2_options_cns = -x ava-ont -t 10 -k17 -w17, and nextgraph_options = -a 1. NextPolish software v.1.2.4: sgs_options = -max_depth 200, lgs_options = -min_read_len 1k -max_read_len 100k -max_depth 100, and lgs_minimap2_options = -x map-ont. ALLHiC software v.0.9.14: “−NonInformativeRatio 0”, “−minREs 50”, “−MaxLinkDensity 3”, “−shortest_ 150”, “−longest_ 800”, “−format_ Sanger”, “−enz DpnII”, and “−CLUSTER 29”. TblastN v.2.2.26: E-value ≤ 1e−5. Swiss-Prot: E-value ≤ 1e−5. Nr: E-value ≤ 1e−5. KEGG: E-value ≤ 1e-3. GO: E-value ≤ 1e-10. Pfam: E-value ≤ 0.01. BLAST to predict rRNAs: E-value ≤ 1e−5, identify ≥ 85%, and match length ≥ 50 bp. BLASTP: E-value ≤ 1e−5. Other commands and pipelines used in data processing were executed using their corresponding default parameters.
